# Pattern of Facial Fractures and Its Association with a Cervical Spine Injury in a Tertiary Hospital in Jordan

**DOI:** 10.1155/2022/4107382

**Published:** 2022-07-30

**Authors:** Fadi Jarab, Anwar Bataineh

**Affiliations:** Department of Oral Medicine and Oral Surgery, Faculty of Dentistry, Jordan University of Science and Technology, P.O. Box 3030, Irbid 22110, Jordan

## Abstract

**Background:**

Facial fractures can be accompanied by serious and life-threatening injuries such as cervical spine injury (CSI), which can lead to serious consequences if misdiagnosed.

**Objective:**

To assess the patterns of maxillofacial fractures and to explore the association between these fractures and cervical spine injuries (CSIs) in patients with a traumatic facial injury.

**Methods:**

A retrospective analysis was conducted on the data of the subjects who were admitted to the King Abdullah University Hospital (KAUH) and had a maxillofacial fracture in the period from January 2017 through December 2020. Stepwise binary logistic regression analysis was conducted to find the variables which are significantly and independently associated with CSIs.

**Results:**

A total of 394 maxillofacial fractures were reported for a total of 221 subjects. The mandible was the most common site of the reported fractures (41.88%). The majority of the subjects had associated injuries (70.6%), of which 82.7% were CSIs. The most common type of the CSIs was the vertebral fracture (52%). Increased age (OR = 1.543, *P* < 0.05), having a mandibular fracture (OR = 4.382, *P* < 0.01), and having a maxillary fracture (OR = 3.269, *P* < 0.05) were significantly associated with the presence of CSI.

**Conclusion:**

The current study revealed that the most common type of facial fracture occurred in the mandible area, and CSI was the most common fracture-associated injury (82.7%). Increased age and having mandibular or maxillary fracture were associated with an increased risk of developing CSI. Therefore, it is necessary to rule out the presence of concomitant CSI during the emergency management of maxillofacial fractures, particularly for elderly patients and those with mandibular or maxillary fractures.

## 1. Introduction

Maxillofacial fractures are traumatic injuries to the face which result in fracturing of the bones of the upper, middle, or lower facial regions [1]. The causes of maxillofacial fractures vary between different age groups and among various communities due to differences in social, economic, and cultural factors [2]. However, road traffic accidents, falls, assaults, sports, and industrial incidents are common causes of maxillofacial fractures development [3]. Other factors such as poor roads and exceeding speed limits can also lead to traffic accidents, and consequently maxillofacial fractures [4, 5]. However, facial trauma caused by high-velocity mechanisms such as motor vehicle accidents are associated with a higher risk for CSI than those caused by falls and workplace accidents [6–8].

Maxillofacial fractures can occur alone or in conjunction with other serious, and sometimes life-threatening injuries to the head, chest, spine, and other regions of the body [9]. Along with other risk factors such as motor vehicle collisions, falls, male gender, and higher injury severity [10, 11], maxillofacial fractures have been recognized as high risk factor for concomitant CSI [6, 12].

Despite the low incidence of CSIs associated with maxillofacial trauma [8, 12–23], their consideration in maxillofacial fractures management is crucial due to the high risk of mortality and neurological complications [24, 25], in addition to its significant physical, psychological, and social impact on the affected patients [26]. Therefore, trauma protocols have emphasized on the importance of the association between maxillofacial fractures and CSI, and the potential negative impact of CSI misdiagnosis [6]. Furthermore, early recognition of the associated CSIs can significantly reduce the morbidity and mortality related to these life-threatening injuries among patients with maxillofacial trauma.

Inconsistent data are available to investigate the association between different facial fractures and the incidence of CSI. Furthermore, the diversity and inconsistency of the factors associated with CSIs necessitate the need for further research to restrict the variability and facilitate the discovery of the genuine predictors of CSIs.

The purpose of this study was to assess the patterns of maxillofacial fractures and to explore the association between these fractures and CSIs in patients who had a traumatic facial injury.

## 2. Methods

### 2.1. Study Design and Subjects

A retrospective analysis was conducted on subjects who had a maxillofacial fracture after being consulted by the neurosurgery team in the period from January 2017 through December 2020 at the King Abdullah University Hospital (KAUH), which is one of the largest hospitals in the north of Jordan, with an operating capacity of 678 beds [27]. The hospital was established to be a high-quality healthcare institution, as well as a comprehensive transformational center for the local and Middle Eastern communities [27].

### 2.2. Data Collection

The data were collected from the medical records of the subjects after receiving plain radiography and computed tomography (CT) scans in order to differentiate between different types of maxillofacial fractures, and the magnetic resonance images in order to explore the potential associated injuries. Patients were enrolled as subjects if they had confirmed facial fractures according to the findings from the radiography and the CT scans. Subjects were excluded from the study sample if their medical record was incomplete or if they had a history of previous maxillofacial fractures. The characteristics of the study subjects such as age, sex, and the different sites of the maxillofacial fractures were the predictor variables in the current study. The primary outcome variable was the incidence of CSI and its associated factors, while the assessment of patterns of maxillofacial fractures was the secondary outcome of the study.

The reason for the injury was classified as a road traffic accident, assault, gunshot, fall, sports, or industrial accident. Maxillofacial fractures were classified as mandibular, maxillary, zygotic, and orbital ones. Mandibular fractures were classified as parasymphysis, angle, condyle, symphysis, body, dentoalveolar, or ramus fractures. Maxillary fractures were classified as LeFort I, LeFort II, LeFort III, or dentoalveolar. Fractures in the zygoma were categorized as zygomatic complex, zygomatic arch fractures, zygomaticomaxillary suture, and zygomaticofrontal suture. Orbital fractures were classified as floor, lateral wall, medial wall, or roof fractures. CSIs were characterized based on the amount of injury confirmed by the radiography, and included vertebral fractures, cervical subluxations, dislocations, disc herniation, cord contusions, and ligament tear. Airway management was classified as no management needed, tracheal intubation, or tracheotomy. Treatment was classified as open reduction and internal fixation, open reduction, closed reduction, or conservative. The study was approved by the Institutional Review Board and the Ethics Committee at KAUH.

### 2.3. Statistical Analysis

Data were analyzed using SPSS, version 27. Categorical data were presented as frequency and percentages. A Chi-square test was conducted to assess the association between the independent variables including age, sex, and the four different sites of the maxillofacial fractures including the mandible, maxilla, zygoma, and the orbit and the dependent variable (CSIs). Variables with a *P* value of < 0.2 were entered into the stepwise binary logistic regression analysis to determine the variables which are significantly and independently associated with the CSIs. A *P* value of < 0.05 was considered statistically significant.

## 3. Results

A total of 394 maxillofacial fractures were reported for a total of 221 subjects, with a mean of 1.78 fractures per subject, and a total of 122 subjects (55.2%) presented with multiple fractures. As shown in Table 1, the mean age (SD) of the subjects was 32 (3.67). Most of the study subjects were males (76.5%) and in the age group of 21 to 30 years (34.9%). A road traffic accident was the most common cause of maxillofacial fractures (62%). As shown in Table 2, the mandibular fracture was the most commonly reported fracture (41.9%), which happened most commonly in the parasymphysis (23.0%) and the angle (22.4%) areas. Nearly 17.8% of the subjects suffered from zygomatic fractures, from which, 48.6% occurred in the zygomaticomaxillary complex and 45.7% were isolated zygomatic arch fractures. As shown in Table 3, the distribution of the maxillary fractures showed that dentoalveolar was the most common fracture of the maxilla (40.6%), followed by the Le Fort I fracture (32.8%). The most common site for orbital fractures was the floor (60.0%). The present study subjects had a total of 156 associated injuries, of which 27 (17.3%) were skull fractures and 129 (82.7%) were CSIs. The most common skull fractures were in the frontal area (66.7%), while vertebral fracture was the most common type of CSIs (52.0%).

Airway management was not indicated in 92.3% of the subjects, while 5.9% and 1.8% of them needed tracheal intubation and required tracheotomy respectively. Results showed no instance of increased neurological impairment as a result of surgical repair of the facial fractures in the present study. The most prevalent treatment technique for maxillofacial fractures was open reduction and internal fixation with plates and other alloplastic materials, accounting for 83.3% of the cases, whereas closed reduction and conservative therapy accounted for the rest of the cases (16.7%).

Variables with a *P* value of < 0.2 from the univariate analysis (Table 4) were entered into the stepwise binary logistic regression analysis. As shown in Table 5, results of the regression analysis showed that each unit increase in age was associated with a 1.543 increase in the odds of having CSI (*P* < 0.05). The odds of having CSI in subjects who suffered from mandibular fracture were 4.382 times the odds of having CSI in subjects who did not have a mandibular fracture (*P* < 0.01). Finally, the odds of having CSI in subjects who suffered from maxillary fracture were 3.269 times the odds of having CSI in subjects who did not have a maxillary fracture (*P* < 0.05).

## 4. Discussion

Limited evidence is available to support the relationship between maxillofacial fractures and CSIs. Therefore, the current study, which is the first one in Jordan, was conducted to assess the patterns of maxillofacial fractures and to explore the association between maxillofacial fractures and CSIs in patients who had a traumatic facial injury.

Consistent with the findings from earlier studies [7, 15, 19, 28], the current study showed a high male:female ratio (3.25 : 1), with the highest prevalence of maxillofacial fractures reported in the age group of 21–30 years. The majority of the current study subjects reported road traffic accidents (62%), followed by falls (14.5%) as the most common causes of maxillofacial fractures. A study conducted in Turkey found that approximately half of the participating subjects had their maxillofacial fracture caused by traffic accidents, and nearly 11.2% of them had fracture caused by falls [28]. Another Pakistani study showed that 46.2% of the facial fractures were caused by road traffic accidents [29]. A systematic review reported that motor vehicle accident was the most common cause of maxillofacial fractures combined with CSI [15], which was consistent with the findings of other studies [8, 21, 23]. This finding could be justified by the fact that the strong force generated by a motor vehicle accident can be distributed from the facial region to the cervical spine area, resulting in a CSI [30].

The present study subjects had a total of 394 maxillofacial fractures, and more than half of them experienced multiple fractures (55.2%). An earlier study reported that nearly 19.5% of the 343 subjects with facial injuries had fractures in different regions of the face [19]. In the present study, the mandible was the most common site of maxillofacial fractures, which is consistent with the findings from earlier studies [7, 8, 13, 29]. Similar results were reported in a study where one-third of the subjects were found to have mandibular fractures in the parasymphysis and angle regions [29]. Another American study reported that 22% of the subjects presented with mandibular fractures, of which, around one-third occurred at the parasymphysis and 43.2% ocuured at the angle sites [16]. This finding could be attributed to the lack of protection on the mandible bone, which is considered the least protected bone in the facial skeleton [31]. On the other hand, only 15% of the subjects enrolled in a US study had a mandibular fracture in the angle site [14]. The present study showed that 17.8% of the maxillofacial fractures were zygomatic fractures. Similarly, 23.6% of the subjects suffered from zygomatic fractures in a study conducted in the US [8]. The zygomatic fractures were almost equally distributed between zygomatic complex fractures (48.57%) and isolated zygomatic arch (45.71%) in the present study. A much lower percentage was reported in an Indian study, where only 8.7% of the facial fractures were identified in the zygomaticomaxillary complex [19]. Another Pakistani study found that around 20% of the subjects had zygomatic bone complex fractures [29]. Regarding maxillary fractures, the most common fracture of the maxilla was the dentoalveolar, followed by the Le Fort I fracture in the present study. In contrast, the most common midface fracture was at the Le Fort I level, followed by the proc. Alveolaris in a Turkish study [28]. However, a recent study found that only 6.5% of the participating subjects had a Le Fort I fracture [29]. Patients with dentoalveolar fractures may not be treated by a surgeon, but rather by a dentist, which may increase the risk of misdiagnosis of a concomitant CSI. Given that missing a CSI can result in severe neurological complications for the patient [25], dentists should refer patients with dentoalveolar fractures to a specialist surgeon in order to rule out the presence of any accompanying CSI, and therefore reducing its negative impact on patients' health. In contrast to the findings from a retrospective review of data of over than 1.3 million trauma subjects, which reported only a 1.6% prevalence of orbital floor fracture [22], the current study reported the floor as the most common site for orbital fractures (60%).

Facial fractures can be associated with concomitant injuries in other parts of the body including the head, spine, abdomen, chest, and limbs, with some of these injuries can be life-threatening [9]. In this study, the majority of the subjects had associated injuries with facial fractures, which was higher than what was found in an African [32] and a Finnish study (25.2%) [9], and less than the findings reported in a US study [33]. Due to the instability of the cervical spine, there is an overreliance on ligamentous structures for stabilization, which makes this part of the spine more susceptible to injuries [34]. The majority of the current study subjects experienced a CSI in association with the facial fracture (82.7%), while only 17.3% of them had an associated skull fracture. On the other hand, head trauma was the most common associated injury in the US [14] and an African study [32], and the second most prevalent associated injury in another study [9]. Compared with the present study finding, the proportion of subjects who suffered from CSI in association with facial trauma was much lower in studies conducted in the US [8, 13, 14, 16, 22, 33], Canada [17], UK [21], Ireland [12], Italy [23], Pakistan [29], India [19], and Africa [32]. In the present study, the frontal bone was the most common site for skull fractures (66.7%). In addition, more than half of the CSIs in this study were vertebral fractures (52%), and 15.5% of them were cervical subluxations. A study conducted in Ireland found that one-third of the subjects who suffered from CSIs had subluxation injuries in the cervical spine [12]. However, subluxation injuries were detected in the majority of the subjects with CSIs in another study [8]. Furthermore, a British study reported that bony fractures, cervical subluxations, and dislocations accounted for 63% of the identified CSIs [21]. The majority of the CSIs occurred at C3–C7 level in the present study (72.9%), which is consistent with the finding from an earlier study conducted in the US (69.5%) [7], while around half of the CSIs occurred at C6–C7 [17, 20, 21], C5–C7 [18], and C4–C7 level [12] in other studies. This finding could be explained by the fact that C3–C7 level is the most frequently injured site in the cervical spine following trauma [35].

In the initial management of patients with suspected CSI, the assessment of the airway status is essential; if the respiratory drive has been lost, thorough airway management utilizing various procedures such as tracheal intubation must be performed to restore oxygen supply to the lungs [36]. Most of the subjects in this study did not require airway management (92.30%), which contradicts what was reported in a British study, where 75% of the subjects required airway control [21]. On the other hand, only 5.9% of the current study subjects needed tracheal intubation, and 1.8% of them required a tracheotomy. A higher percentage was reported in a study conducted in the UK, where more than one-third of the subjects who had facial fractures with concomitant CSI underwent a tracheostomy or tracheal intubation during admission [21]. Consistent with the findings from an earlier study [21], all subjects who had both maxillofacial and CSIs were treated with collars, halo braces, sandbags, and tapes for intraoperative head and neck immobilization in the present study. Similarly, almost all of the participating subjects with CSI were treated conservatively with collar immobilization in other studies [20, 23]. Additionally, there was no instance of increased neurological impairment as a result of surgical repair of the facial fractures in the current study. Similar results were reported in previous studies [20, 21]. The most common treatment modality in this study was open reduction and internal fixation with plates and other alloplastic materials (83.3%). However, open reduction was used in a small number of subjects with a CSI enrolled in a Turkish study [28]. Although 40.9% of the subjects who participated in a Greek study performed open reduction and internal fixation, open reduction without internal fixation was used in only one subject [20]. On the other hand, closed reduction and conservative treatment were performed in only 16.7% of the cases in the present study.

When exploring the factors associated with CSI in the present study, the results showed that increased age was associated with an increased risk of developing CSI. This finding is consistent with that of previous studies [9, 14, 37] and contradicts the finding from an African study [32]. Furthermore, having a mandibular or a maxillary fracture was associated with having CSI in the current study. A study conducted in the UK reported a statistically significant difference in the incidence of CSI between subjects who had maxillofacial fractures versus those who had not [21]. Similarly, an African study found that associated injuries were significantly more observed in subjects who had mandibular fractures when compared with those who had other types of fractures [32]. In contrast, logistic regression results of a study conducted in Finland showed that associated injuries were observed more often in subjects who had zygomatico-orbital, severe midfacial, or combination fractures than those with exclusively mandibular fractures [9].

### 4.1. Study Limitations

The study was conducted in the north of Jordan and not across the country, which could limit the number of the tested cases. Furthermore, the Jordanian government implemented strict traffic legislation that could have limited the number of traffic accidents in Jordan, and subsequently, the number of facial fractures cases reported in this study and increasing the sample size would allow for more robust conclusions to be withdrawn from the study. However, similar samples were included in earlier research conducted in Turkey [28] and the US [33].

## 5. Conclusion

Mandibular fractures represented 60% of the maxillofacial fractures identified in the current study. The incidence of the associated injuries occurred in 70.6% of the fractures, with 82.7% of these injuries occurring in the cervical spine area. In addition to increased age, having mandibular fractures or fractures in the maxilla region was associated with an increased risk of CSI in the present study.

## Figures and Tables

**Table 1:** Characteristics of the study subjects (*n* = 221).

Characteristics	Frequency	Percentage
Age group		
≤10	19	9.07
11–20	51	22.62
21–30	76	34.39
31–40	47	21.26
41–50	17	7.69
>50	11	4.97

Sex		
Male	169	76.5
Female	52	23.5

Causes of maxillofacial fracture		
Road traffic accidents	137	62.0
Assault	29	13.1
Gunshot	5	2.3
Falls	32	14.5
Sports	10	4.5
Industrial accidents	8	3.6

**Table 2:** Distribution of the anatomic site of the maxillofacial fractures.

Maxillofacial fractures	*N* (%)	Mandibular fractures	*N* (%)	Maxilla fractures	*N* (%)	Zygoma fractures	*N* (%)	Orbital fractures	*N* (%)
Mandible	165 (41.88)	Angle	37 (22.42)	Le Fort 1	21 (32.8)	Zygomaticomaxillary suture	1 (1.4)	Floor	57 (60)
Maxilla	64 (16.24)	Parasymphysis	38 (23.03)	Dentoalveolar	26 (40.6)	Isolated zygomatic arch	32 (45.71)	Lateral wall	18 (18.94)
Zygoma	70 (17.77)	Body	32 (19.39)	Le Fort II	10 (15.6)	Zygomaticofrontal suture	3 (4.28)	Medial wall	11 (11.57)
Orbital	95 (24.11)	Dentoalveolar	20 (12.12)	Le Fort III	7 (10.9)	Zygomatic complex	34 (48.57)	Roof	9 (9.47)
Total	394 (100)	Condyle	16 (9.69)	Total	64 (100)	Total	70 (100)	Total	95 (100)
		Ramus	4 (2.2)						
		Symphysis	18 (10.91)						
		Total	165 (100)						

**Table 3:** Distribution of cervical spine injury among the study subjects.

Cervical spine injury	*N* (%)
Cervical subluxations	20 (15.5)
Dislocations	10 (7.8)
Disc herniation	8 (6.2)
Cord contusions	13 (10.0)
Ligament tear	11 (8.5)
Vertebral fracture	67 (52.0)
Total	129 (100)

C-level fracture	*N* (%)
C1	25 (19.4)
C2	10 (7.7)
C3–C7	94 (72.9)
Total	129 (100)

**Table 4:** Univariate analysis of the variables associated with cervical spine injuries.

Variable	Cervical spine injury		*P* value
	Absent	Present	
Age (mean (SD))	22 (2.9)	38 (3.9)	0.126^*∗*^
	Frequency (Percentage %)		
Sex (male)	92 (54.4)	77 (45.6)	0.395
Having mandibular fractures	69 (41.8)	96 (58.2)	0.054^*∗*^
Having maxillary fractures	22 (34.4)	42 (65.6)	0.029^*∗*^
Having zygomatic fractures	27 (38.6)	43 (61.4)	0.034^*∗*^
Having orbital fractures	42 (44.2)	53 (55.8)	0.238

^*∗*^Variables with *P* value < 0.2.

**Table 5:** Multivariate analysis of the variables associated with cervical spine injuries.

Variable		OR (95% confidence interval)	*P* value
Having mandibular fracture	No	Reference	0.007^*∗∗*^
	Yes	4.382 (2.273–7.692)	

Having maxilla fracture	No	Reference	0.023^*∗*^
	Yes	3.269 (2.142–4.944)	

Age		1.543 (1.10–2.259)	0.039^*∗*^

^*∗*^Significant at 0.05 level; ^*∗∗*^significant at 0.01 level.

## Data Availability

The data are available at the corresponding author and could be provided upon request.
